# Applying a cumulative deficit model of frailty to dementia: progress and future challenges

**DOI:** 10.1186/s13195-014-0084-z

**Published:** 2014-11-26

**Authors:** Kaarin J Anstey, Roger A Dixon

**Affiliations:** Centre for Research on Ageing, Health and Wellbeing, Australian National University, 63 Eggleston Road, Canberra, ACT 0200 Australia; Department of Psychology, P217 Biological Sciences Building, University of Alberta, Edmonton, AB T6G 2E9 Canada

## Abstract

The article by Song and colleagues presents findings from the Canadian Study of Health and Aging showing that the accumulation of health deficits, defined dichotomously and unqualified by severity or domain, predicted late-life dementia independent of chronological age. We identify strengths of this model, and also areas for future research. Importantly, this article broadens the perspective of research into measuring risk of dementia from focusing on specific neuropathological markers of dementia subtypes, to mechanisms underlying more general bodily vitality and health, as well as dysfunctions in repair. This work places late-life dementia in a new context, influenced more broadly by health maintenance, and less by specific neurological disease. While useful at a global level, the lack of specificity of this approach may ultimately limit its application to individual patients because without linking risk to etiology, assessment does not indicate an intervention. Ultimately, the article has value for stimulating debate about approaches to risk identification and risk reduction, suggesting that the current focus on cardiometabolic risk factors may be too limited.

## Commentary

In their recent article Song and colleagues [1] present findings from the Canadian Study of Health and Aging showing that the accumulation of health deficits, defined dichotomously and unqualified by severity or domain, predicted late-life dementia independent of chronological age. Many of the deficits included in their statistical models were not traditional or specific dementia risk factors (for example, vascular), but were health problems related to various conditions associated with skin, ears, eyes, foot, nose and even attitudes. Such health problems have been used previously in accumulation indices of frailty [2] and dementia [3]. The rigorous statistical models applied demonstrate that these associations were robust findings [3,4].

The authors’ programmatic work makes several contributions to epidemiology of dementia. They demonstrate the predictive value of considering multiple health deficits in the aggregate, and that various combinations predict adverse outcomes, including falls, delirium, disability, and now dementia [1-3,5]. An important conclusion is that overall health status is a major or contributing risk factor for dementia. Although specific mechanisms are deliberately not evaluated in this work, the authors propose that the generality of associations of poor health with dementia may be related to impaired repair mechanisms, or exhausted repair responses. These deficient processes manifest across multiple domains of health and are not necessarily or directly related to neurological function.

We turn now to brief considerations of selected conceptual, methodological, and clinical challenges for future progress in this important line of research. The general proposition of the authors [1] is that the accumulation of health deficits provides an index of general health. However, there is a potential conceptual circularity to the explanation if general health is defined simply (and dichotomously) by the presence or absence of physical health problems and feelings of vitality and energy. Would additional conceptual clarity be achieved by consideration of severities, durations, clusters, or weightings of conditions (along a continuum from the proximal to distal *vis-à-vis* dementia)? Also absent from the accumulation of deficits index is any temporal ordering of deficits. Temporal ordering is a feature of cascade theories of ageing in which accumulation of deficits occur in a general sequence, potentially leading to an acceleration of decline once a threshold is reached. The most prominent example of a cascade theory is the amyloid hypothesis of Alzheimer’s disease [6].

Song and colleagues’ index [1] focuses on deficits, in contrast to indices of functional capacity that include measures of capacity or biological health, referred to as ‘Bioage’. Bioage indices typically weight the component measures, and represent general and available biological functions and ability. They have been linked to a variety of adverse outcomes (for example, cognitive decline, longevity), comparing favorably with chronological age [7].

Although a health status index predicts important adverse outcomes (from falls to dementia), the authors acknowledge theoretical and clinical limitations. Framed as challenges for future development, these include the notion that dementia is a broad clinical outcome produced by underlying but heterogeneous neuropathological pathways. The mechanisms associated with dementia are likely to differ depending on underlying neurodegenerative disease processes. The cumulative health deficit approach is remarkably successful at predicting dementia, but dementia *per se* may not be the principal outcome of interest theoretically or clinically (in terms of interventions). Whereas it is true (as the authors point out) that single or candidate risk factors are also unlikely to produce good status predictions or unqualified insights into mechanisms, the dementia biomarker and risk factor field is moving rapidly to close the gap between the single and the multiple markers and moderators of sporadic neurodegenerative disease. Contemporary approaches include additive (similar to the authors) or panel models, but also multiplicative (interactional) models, multiple and cross-domain approaches, and even broad-based OMICs-type approaches (linking the global and specific) [8]. A single dementia predictive index of health deficits does not allow for specificity in relation to the type of dementia or the etiology of cognitive impairment. Such specific information may be useful, if not crucial, in devising interventions to delay the onset of dementia because intervention work requires some degree of knowledge about mechanisms. In the future, the deficit accumulation approach may benefit from coordinating information that provides specificity or discrimination of dementias. Much current research into biomarkers and risk factors for dementia focuses on identifying biomarkers that relate to specific disease processes [9-11] which will ultimately inform diagnosis and treatment. Hence, their eventual utility may be greater than (but supplemented by) a generic index.

To promote understanding of the statistical associations between this index and dementia, a tangible biological mechanism or biomarker of the repair mechanism underlying health failures is required. The significance of this index could be better understood by estimating the variance it explains that is either shared with, or independent of, traditional risk factors or indices that have been developed from these. Demonstration of the utility of the index in identifying preclinical cognitive decline would increase its significance. At present, the authors’ approach could be enhanced by a focus on early detection and selective diagnoses, as this preclinical period is likely the one most promising for specific interventions.

## Conclusion

Importantly, Song and colleagues [1] broaden the perspective of research into measuring risk of dementia from focusing on specific causes of neuropathological markers of dementia subtypes, to mechanisms underlying more general bodily vitality and health, as well as dysfunctions in repair. This places late-life dementia in a new context, influenced more broadly by health maintenance, and less by specific neurological disease. This approach may benefit from further integration - rather than separation - into the emerging multi-variable and mechanism-related approaches to biomarker and risk factor research. An intriguing question is whether the identification of a poor repair mechanism would explain the occurrence of the individual risk factors (or vice versa). It would also be interesting to know how the frailty index relates to genetic markers of longevity (and neurodegenerative disease). Most importantly, it would be useful to discover whether an intervention to improve ‘repair mechanisms’ could have widespread benefits across multiple cognitive health conditions or is better supplemented by specific mechanism-related therapeutics.
